# Supplementary material for: End Sequence Analysis Toolkit (ESAT) expands the extractable information from single-cell RNA-seq data
Source: Genome Res. 2016 Oct;26(10):1397–410. doi: 10.1101/gr.207902.116 (PMC5052061; doi:10.1101/gr.207902.116)
Supplement: Supplemental Material [file supp_gr.207902.116_Supp_Materials.tar.gz › ESAT-esat_v0.1/doc/html/classbroad_1_1core_1_1sequence_1_1_fasta_sequence_i_o.html]

ESAT: broad.core.sequence.FastaSequenceIO Class Reference


ESAT


- Main Page
- Related Pages
- Packages
- Classes
- Files

- Class List
- Class Index
- Class Hierarchy
- Class Members

All Classes Namespaces Files Functions Variables Enumerator Pages

- broad
- core
- sequence
- FastaSequenceIO

Public Member Functions |
Static Public Member Functions |
Static Public Attributes |
Protected Member Functions |
List of all members

broad.core.sequence.FastaSequenceIO Class Reference

Inheritance diagram for broad.core.sequence.FastaSequenceIO:

[legend]

Collaboration diagram for broad.core.sequence.FastaSequenceIO:

[legend]

|  |  |
| --- | --- |
| Public Member Functions | |
|  | FastaSequenceIO (String fileName) |
|  | |
|  | FastaSequenceIO (File file) |
|  | |
|  | FastaSequenceIO () |
|  | |
| List< Sequence > | loadAll () throws IOException |
|  | |
| List< Sequence > | loadAll (InputStream is) throws IOException |
|  | |
| void | extractRecordsIntoSequenceList (List<?extends Sequence > sequenceList) throws Exception |
|  | |
| void | mergeRecords (Sequence seq, String regEx) throws IOException |
|  | |
| void | writeRecordsWithMinLength (int minimumLength, InputStream inIs, BufferedWriter bw) throws IOException |
|  | |
| List< Sequence > | extractRecordsWithIDsMatching (List< String > regExs, boolean encode) throws IOException |
|  | |
| List< Sequence > | extractRecordsWithIDsMatching (List< String > regExs, boolean encode, int expectedSize) throws IOException |
|  | |
| List< Sequence > | extractRecordsWithIDLike (String regEx, boolean encode) throws IOException |
|  | |
| List< Sequence > | extractRecordsWithIDLike (String regEx, boolean encode, int expectedSize) throws IOException |
|  | |
| List< Sequence > | extractRecords (Collection< String > recordIdList, InputStream is) throws IOException |
|  | |
| List< Sequence > | extractRecords (Collection< String > recordIdList, InputStream is, boolean largeSequences) throws IOException |
|  | |
| List< Sequence > | extractRecords (Collection< String > recordIdList) throws IOException |
|  | |
| void | extractRegion (SequenceRegion region) throws IOException |
|  | |
| void | extractRegions (List<?extends SequenceRegion > regions) throws IOException |
|  | |
| void | extractRegions (List<?extends SequenceRegion > regions, boolean useRegionOrientation) throws IOException |
|  | |
| void | extractRegions (List<?extends SequenceRegion > regions, boolean useRegionOrientation, InputStream is) throws IOException |
|  | |
| void | write (List<?extends Sequence > seqs, int lineLength) throws IOException |
|  | |
| void | write (List<?extends Sequence > seqs) throws IOException |
|  | |
| void | write (Sequence seq) throws IOException |
|  | |
| void | write (Sequence seq, String fileName) throws IOException |
|  | |
| void | append (List<?extends Sequence > records, String fileName) throws IOException |
|  | |
| void | append (Sequence seq, String output) throws IOException |
|  | |
| void | write (List<?extends Sequence > seqs, String fileName) throws IOException |
|  | |
| void | write (List<?extends Sequence > seqs, String fileName, int lineLength) throws IOException |
|  | |
| void | write (List<?extends Sequence > seqs, BufferedWriter bw, int lineLength) throws IOException |
|  | |
| void | write (List<?extends Sequence > seqs, BufferedWriter bw) throws IOException |
|  | |
| void | write (List<?extends Sequence > seqs, BufferedWriter bw, Map< String, String > names, String refID, String pos) throws IOException |
|  | |
| void | write (Sequence seq, BufferedWriter bw, int lineLength) throws IOException |
|  | |
| void | write (Sequence seq, BufferedWriter bw) throws IOException |
|  | |
| void | unchunk (File[] inDirList, int chunkSize, String unchunkSeqName, String outputFile) throws IOException |
|  | |
| void | setSource (File newSource) |
|  | |
| void | breakUpMultifile (final int sizeToBreakup) throws IOException |
|  | |

|  |  |
| --- | --- |
| Static Public Member Functions | |
| static void | main (String[] args) throws IOException |
|  | |
| static Collection< Sequence > | loadSequences (File fasta) throws IOException |
|  | |
| static Map< String, Sequence > | loadSequencesByNameFromDirectory (File sequenceDirectory) throws Exception |
|  | |
| static Map< String, Sequence > | loadSequencesByNameFromDirectory (File sequenceDirectory, boolean nonrandomOnly) throws Exception |
|  | |
| static Collection< String > | getSequenceNames (String fasta) throws IOException |
|  | |
| static Map< String, Sequence > | getChrSequencesFromFasta (String genomeFasta) throws IOException |
|  | |
| static String | createSizeFile (String refFasta) throws IOException |
|  | |

|  |  |
| --- | --- |
| Static Public Attributes | |
| static final int | LINE\_LENGTH = 60 |
|  | |

|  |  |
| --- | --- |
| Protected Member Functions | |
| void | writeSequenceId (Sequence seq, BufferedWriter bw) throws IOException |
|  | |

## Detailed Description

Suppose to be a very generic and smart Fasta manipulation Class. It is meant to handle both small and very large, multi and single fasta files.

As ussual its initial version will be soooo much less ambitious.

Author
:   MGarber

## Constructor & Destructor Documentation

|  |  |  |  |  |  |
| --- | --- | --- | --- | --- | --- |
| broad.core.sequence.FastaSequenceIO.FastaSequenceIO | ( | String | *fileName* | ) |  |

|  |  |  |  |  |  |
| --- | --- | --- | --- | --- | --- |
| broad.core.sequence.FastaSequenceIO.FastaSequenceIO | ( | File | *file* | ) |  |

|  |  |  |  |  |
| --- | --- | --- | --- | --- |
| broad.core.sequence.FastaSequenceIO.FastaSequenceIO | ( |  | ) |  |

Here is the caller graph for this function:

## Member Function Documentation

|  |  |  |  |
| --- | --- | --- | --- |
| void broad.core.sequence.FastaSequenceIO.append | ( | List<?extends Sequence > | *records*, |
|  |  | String | *fileName* |
|  | ) |  | throws IOException |

Here is the call graph for this function:

Here is the caller graph for this function:

|  |  |  |  |
| --- | --- | --- | --- |
| void broad.core.sequence.FastaSequenceIO.append | ( | Sequence | *seq*, |
|  |  | String | *output* |
|  | ) |  | throws IOException |

Here is the call graph for this function:

|  |  |  |  |  |  |
| --- | --- | --- | --- | --- | --- |
| void broad.core.sequence.FastaSequenceIO.breakUpMultifile | ( | final int | *sizeToBreakup* | ) | throws IOException |

|  |  |  |  |  |  |
| --- | --- | --- | --- | --- | --- |
| static String broad.core.sequence.FastaSequenceIO.createSizeFile | ( | String | *refFasta* | ) | throws IOException |

static

Here is the call graph for this function:

|  |  |  |  |
| --- | --- | --- | --- |
| List<Sequence> broad.core.sequence.FastaSequenceIO.extractRecords | ( | Collection< String > | *recordIdList*, |
|  |  | InputStream | *is* |
|  | ) |  | throws IOException |

Here is the caller graph for this function:

|  |  |  |  |
| --- | --- | --- | --- |
| List<Sequence> broad.core.sequence.FastaSequenceIO.extractRecords | ( | Collection< String > | *recordIdList*, |
|  |  | InputStream | *is*, |
|  |  | boolean | *largeSequences* |
|  | ) |  | throws IOException |

|  |  |  |  |  |  |
| --- | --- | --- | --- | --- | --- |
| List<Sequence> broad.core.sequence.FastaSequenceIO.extractRecords | ( | Collection< String > | *recordIdList* | ) | throws IOException |

Here is the call graph for this function:

|  |  |  |  |  |  |
| --- | --- | --- | --- | --- | --- |
| void broad.core.sequence.FastaSequenceIO.extractRecordsIntoSequenceList | ( | List<?extends Sequence > | *sequenceList* | ) | throws Exception |

|  |  |  |  |
| --- | --- | --- | --- |
| List<Sequence> broad.core.sequence.FastaSequenceIO.extractRecordsWithIDLike | ( | String | *regEx*, |
|  |  | boolean | *encode* |
|  | ) |  | throws IOException |

|  |  |  |  |
| --- | --- | --- | --- |
| List<Sequence> broad.core.sequence.FastaSequenceIO.extractRecordsWithIDLike | ( | String | *regEx*, |
|  |  | boolean | *encode*, |
|  |  | int | *expectedSize* |
|  | ) |  | throws IOException |

Here is the call graph for this function:

|  |  |  |  |
| --- | --- | --- | --- |
| List<Sequence> broad.core.sequence.FastaSequenceIO.extractRecordsWithIDsMatching | ( | List< String > | *regExs*, |
|  |  | boolean | *encode* |
|  | ) |  | throws IOException |

Here is the caller graph for this function:

|  |  |  |  |
| --- | --- | --- | --- |
| List<Sequence> broad.core.sequence.FastaSequenceIO.extractRecordsWithIDsMatching | ( | List< String > | *regExs*, |
|  |  | boolean | *encode*, |
|  |  | int | *expectedSize* |
|  | ) |  | throws IOException |

|  |  |  |  |  |  |
| --- | --- | --- | --- | --- | --- |
| void broad.core.sequence.FastaSequenceIO.extractRegion | ( | SequenceRegion | *region* | ) | throws IOException |

Here is the call graph for this function:

|  |  |  |  |  |  |
| --- | --- | --- | --- | --- | --- |
| void broad.core.sequence.FastaSequenceIO.extractRegions | ( | List<?extends SequenceRegion > | *regions* | ) | throws IOException |

Here is the caller graph for this function:

|  |  |  |  |
| --- | --- | --- | --- |
| void broad.core.sequence.FastaSequenceIO.extractRegions | ( | List<?extends SequenceRegion > | *regions*, |
|  |  | boolean | *useRegionOrientation* |
|  | ) |  | throws IOException |

Extracts sequence regions from fasta file into

Parameters
:   |  |  |
    | --- | --- |
    | regions |  |
    | useRegionOrientation | if true it reverses the sequence extracted. |

Exceptions
:   |  |  |
    | --- | --- |
    | IOException |  |

Here is the call graph for this function:

|  |  |  |  |
| --- | --- | --- | --- |
| void broad.core.sequence.FastaSequenceIO.extractRegions | ( | List<?extends SequenceRegion > | *regions*, |
|  |  | boolean | *useRegionOrientation*, |
|  |  | InputStream | *is* |
|  | ) |  | throws IOException |

Extracts sequence regions from fasta file into

Parameters
:   |  |  |
    | --- | --- |
    | regions |  |
    | useRegionOrientation | if true it reverses the sequence extracted. |

Exceptions
:   |  |  |
    | --- | --- |
    | IOException |  |

Here is the call graph for this function:

|  |  |  |  |  |  |
| --- | --- | --- | --- | --- | --- |
| static Map<String, Sequence> broad.core.sequence.FastaSequenceIO.getChrSequencesFromFasta | ( | String | *genomeFasta* | ) | throws IOException |

static

Parameters
:   |  |  |
    | --- | --- |
    | genomeFasta | Fasta file of chromosomes |

Returns
:   Map of chromosome name to sequence

Exceptions
:   |  |  |
    | --- | --- |
    | IOException |  |

Here is the call graph for this function:

Here is the caller graph for this function:

|  |  |  |  |  |  |
| --- | --- | --- | --- | --- | --- |
| static Collection<String> broad.core.sequence.FastaSequenceIO.getSequenceNames | ( | String | *fasta* | ) | throws IOException |

static

|  |  |  |  |  |
| --- | --- | --- | --- | --- |
| List<Sequence> broad.core.sequence.FastaSequenceIO.loadAll | ( |  | ) | throws IOException |

|  |  |  |  |  |  |
| --- | --- | --- | --- | --- | --- |
| List<Sequence> broad.core.sequence.FastaSequenceIO.loadAll | ( | InputStream | *is* | ) | throws IOException |

|  |  |  |  |  |  |
| --- | --- | --- | --- | --- | --- |
| static Collection<Sequence> broad.core.sequence.FastaSequenceIO.loadSequences | ( | File | *fasta* | ) | throws IOException |

static

Here is the call graph for this function:

|  |  |  |  |  |  |
| --- | --- | --- | --- | --- | --- |
| static Map<String, Sequence> broad.core.sequence.FastaSequenceIO.loadSequencesByNameFromDirectory | ( | File | *sequenceDirectory* | ) | throws Exception |

static

|  |  |  |  |
| --- | --- | --- | --- |
| static Map<String, Sequence> broad.core.sequence.FastaSequenceIO.loadSequencesByNameFromDirectory | ( | File | *sequenceDirectory*, |
|  |  | boolean | *nonrandomOnly* |
|  | ) |  | throws Exception |

static

|  |  |  |  |  |  |
| --- | --- | --- | --- | --- | --- |
| static void broad.core.sequence.FastaSequenceIO.main | ( | String[] | *args* | ) | throws IOException |

static

Here is the call graph for this function:

|  |  |  |  |
| --- | --- | --- | --- |
| void broad.core.sequence.FastaSequenceIO.mergeRecords | ( | Sequence | *seq*, |
|  |  | String | *regEx* |
|  | ) |  | throws IOException |

|  |  |  |  |  |  |
| --- | --- | --- | --- | --- | --- |
| void broad.core.sequence.FastaSequenceIO.setSource | ( | File | *newSource* | ) |  |

|  |  |  |  |
| --- | --- | --- | --- |
| void broad.core.sequence.FastaSequenceIO.unchunk | ( | File[] | *inDirList*, |
|  |  | int | *chunkSize*, |
|  |  | String | *unchunkSeqName*, |
|  |  | String | *outputFile* |
|  | ) |  | throws IOException |

Here is the call graph for this function:

|  |  |  |  |
| --- | --- | --- | --- |
| void broad.core.sequence.FastaSequenceIO.write | ( | List<?extends Sequence > | *seqs*, |
|  |  | int | *lineLength* |
|  | ) |  | throws IOException |

Here is the caller graph for this function:

|  |  |  |  |  |  |
| --- | --- | --- | --- | --- | --- |
| void broad.core.sequence.FastaSequenceIO.write | ( | List<?extends Sequence > | *seqs* | ) | throws IOException |

Here is the call graph for this function:

|  |  |  |  |  |  |
| --- | --- | --- | --- | --- | --- |
| void broad.core.sequence.FastaSequenceIO.write | ( | Sequence | *seq* | ) | throws IOException |

Here is the call graph for this function:

|  |  |  |  |
| --- | --- | --- | --- |
| void broad.core.sequence.FastaSequenceIO.write | ( | Sequence | *seq*, |
|  |  | String | *fileName* |
|  | ) |  | throws IOException |

Here is the call graph for this function:

|  |  |  |  |
| --- | --- | --- | --- |
| void broad.core.sequence.FastaSequenceIO.write | ( | List<?extends Sequence > | *seqs*, |
|  |  | String | *fileName* |
|  | ) |  | throws IOException |

Here is the call graph for this function:

|  |  |  |  |
| --- | --- | --- | --- |
| void broad.core.sequence.FastaSequenceIO.write | ( | List<?extends Sequence > | *seqs*, |
|  |  | String | *fileName*, |
|  |  | int | *lineLength* |
|  | ) |  | throws IOException |

Here is the call graph for this function:

|  |  |  |  |
| --- | --- | --- | --- |
| void broad.core.sequence.FastaSequenceIO.write | ( | List<?extends Sequence > | *seqs*, |
|  |  | BufferedWriter | *bw*, |
|  |  | int | *lineLength* |
|  | ) |  | throws IOException |

Here is the call graph for this function:

|  |  |  |  |
| --- | --- | --- | --- |
| void broad.core.sequence.FastaSequenceIO.write | ( | List<?extends Sequence > | *seqs*, |
|  |  | BufferedWriter | *bw* |
|  | ) |  | throws IOException |

Here is the call graph for this function:

|  |  |  |  |
| --- | --- | --- | --- |
| void broad.core.sequence.FastaSequenceIO.write | ( | List<?extends Sequence > | *seqs*, |
|  |  | BufferedWriter | *bw*, |
|  |  | Map< String, String > | *names*, |
|  |  | String | *refID*, |
|  |  | String | *pos* |
|  | ) |  | throws IOException |

Here is the call graph for this function:

|  |  |  |  |
| --- | --- | --- | --- |
| void broad.core.sequence.FastaSequenceIO.write | ( | Sequence | *seq*, |
|  |  | BufferedWriter | *bw*, |
|  |  | int | *lineLength* |
|  | ) |  | throws IOException |

Here is the call graph for this function:

|  |  |  |  |
| --- | --- | --- | --- |
| void broad.core.sequence.FastaSequenceIO.write | ( | Sequence | *seq*, |
|  |  | BufferedWriter | *bw* |
|  | ) |  | throws IOException |

Here is the call graph for this function:

|  |  |  |  |
| --- | --- | --- | --- |
| void broad.core.sequence.FastaSequenceIO.writeRecordsWithMinLength | ( | int | *minimumLength*, |
|  |  | InputStream | *inIs*, |
|  |  | BufferedWriter | *bw* |
|  | ) |  | throws IOException |

Here is the call graph for this function:

|  |  |  |  |
| --- | --- | --- | --- |
| void broad.core.sequence.FastaSequenceIO.writeSequenceId | ( | Sequence | *seq*, |
|  |  | BufferedWriter | *bw* |
|  | ) |  | throws IOException |

protected

Here is the caller graph for this function:

## Member Data Documentation

final int broad.core.sequence.FastaSequenceIO.LINE\_LENGTH = 60

static

---

The documentation for this class was generated from the following file:

- java/broad/core/sequence/FastaSequenceIO.java


---

Generated on Fri Aug 15 2014 15:10:56 for ESAT by  

 1.8.7
